# Non-visual homing and the current status of navigation in scorpions

**DOI:** 10.1007/s10071-020-01386-z

**Published:** 2020-04-29

**Authors:** Emily Danielle Prévost, Torben Stemme

**Affiliations:** Institute of Neurobiology, University of Ulm, Albert-Einstein-Allee 11, 89081 Ulm, Germany

**Keywords:** Path integration, Home vector, Proprioception, Chelicerata, Spatial cognition, Mechanosensation

## Abstract

Within arthropods, the investigation of navigational aspects including homing abilities has mainly focused on insect representatives, while other arthropod taxa have largely been ignored. As such, scorpions are rather underrepresented concerning behavioral studies for reasons such as low participation rates and motivational difficulties. Here, we review the sensory abilities of scorpions related to navigation. Furthermore, we present an improved laboratory setup to shed light on navigational abilities in general and homing behavior in particular. We tracked directed movements towards home shelters of the lesser Asian scorpion *Mesobuthus eupeus* to give a detailed description of their departure and return movements. To do so, we analyzed the departure and return angles as well as measures of directness like directional deviation, lateral displacement, and straightness indices. We compared these parameters under different light conditions and with blinded scorpions. The motivation of scorpions to leave their shelter depends strongly upon the light condition and the starting time of the experiment; highest participation rates were achieved with infrared conditions or blinded scorpions, and close to dusk. Naïve scorpions are capable of returning to a shelter object in a manner that is directionally consistent with the home vector. The first-occurring homing bouts are characterized by paths consisting of turns about 10 cm to either side of the straightest home path and a distance efficiency of roughly three-quarters of the maximum efficiency. Our results show that neither chemosensation nor vision, but rather path integration based on proprioception, plays a superior role in the homing of scorpions.

**Electronic supplementary material:**

The online version of this article (10.1007/s10071-020-01386-z) contains supplementary material, which is available to authorized users.

## Introduction and review

The Arthropoda, covering such diverse taxa as insects, crustaceans, centipedes, spiders, and their kin, are famous for their exceptional navigational abilities. Since its establishment as an active field of science, the investigation of navigation and orientation, often in close relation to terms like learning and memory, has fascinated scientists and laypersons alike. The deciphering of the bee’s waggle dance and the superb navigational abilities of desert ants are only two examples (von Frisch and Lindauer 1956; Wehner 2003).

One specific branch of navigation research deals with homing behavior, which is defined as the ability of individuals to return to a fixed location after an excursion therefrom (e.g., Warrant and Dacke 2010). The earliest studies on homing behavior in arthropods were performed approximately 150 years ago by displacing hymenopteran insects (Fabre 1879, 1882). As in many aspects, our knowledge of orientation and homing behavior is based on several model organisms, and conclusions obtained are often extrapolated to the entire taxon to which the model organisms belong, or even to the entire arthropod phylum. In the context of arthropod behavior, a strong bias exists toward insects in general, and hymenopterans in particular. Other taxa outside the insects have received limited attention so far. Although some knowledge has been gained for a few representatives of chelicerates (reviewed in Wehner 1992), our understanding of their navigational abilities remains rather fragmented. Nevertheless, some excellent work has accumulated in recent years on the navigation and homing of spiders (Dacke et al. 1999; Nørgaard et al. 2003, 2012), whip spiders (Bingman et al. 2017; Wiegmann et al. 2019), and harvestmen (Silva et al. 2018). Other chelicerate taxa remain underrepresented, including the arachnid order of scorpions.

### Sensory abilities of scorpions

#### Mechanosensation

**Fig. 1:**
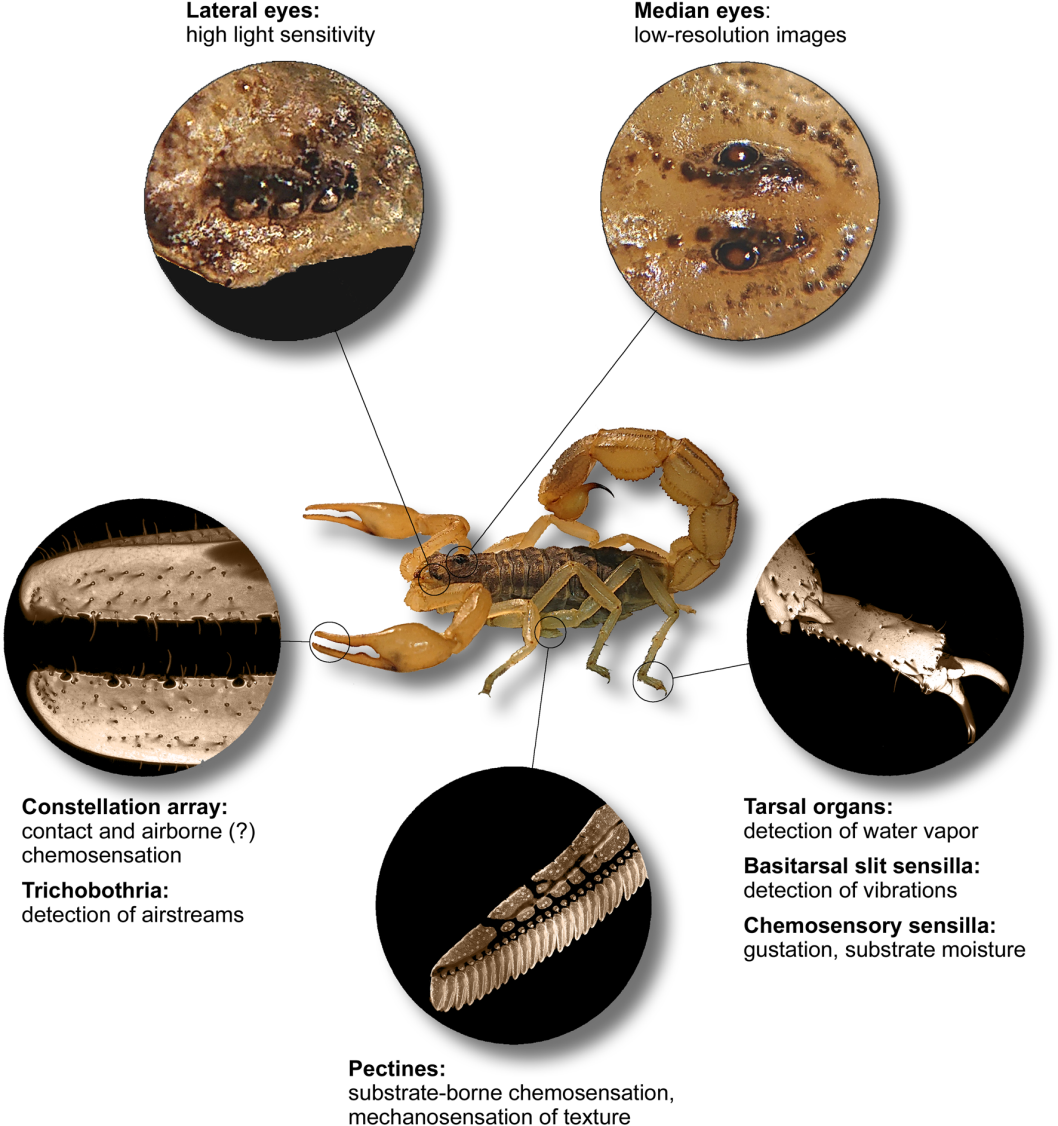
The primary sensory organs in the scorpion *Mesobuthus eupeus* (photos: originals) and their functional abilities gathered from the literature on scorpion sensation (see “Introduction and review” for detailed information and references)

Scorpions show an impressive repertoire of sensory systems detecting a multitude of stimuli (Fig. 1) which might be associated with their navigational and homing abilities. These include mechanosensory hairs or trichobothria distributed all over the body, especially on the pedipalps. The sensory hairs and trichobothria react to air streams and possess directional sensitivity (Hoffmann 1967; Linsenmair 1968, 1972; Fleissner 1977a, b; Krapf 1987; Meßlinger 1987; Fleissner and Fleissner 2001a), and might also facilitate detection of substrate vibrations (Brownell and Farley 1979a, b, c). Other mechanosensory receptors, namely slit organs, have been described near the leg joins of scorpions (Pringle 1955; Barth and Wadepuhl 1975; Barth and Stagl 1976). The basitarsal slit sensilla function as vibration detectors in sand scorpions (Brownell and van Hemmen 2001). Although it has never been demonstrated, the slit organs on other leg joints in scorpions might be associated with proprioception, as it has been shown for other arachnids (e.g., Seyfarth and Barth 1972; Seyfarth et al. 1982). Scorpions use their mechanical sense to stay in contact with physical objects. They exhibit negative thigmokinesis (slowing or stopping their movement when in lateral contact with an object) and positive thigmotaxis (directing their movements toward an object with which they have come in contact) (Abushama 1964). In addition, disturbed scorpions seek dorsal contact in the escape response (Torres and Heatwole 1967). Therefore, physical contact probably plays an important role in finding the entrance of a shelter and perhaps selecting a suitable shelter.

#### Chemosensation

Another sensory modality which has been studied in great detail is chemosensation. Chelicerata in general and scorpions in particular do not possess antennae or other chemosensory appendages associated with the head. Scorpions instead possess so-called pectines as dedicated pairs of chemosensory appendages, which are studded with thousands of chemosensory sensilla and also fulfill a mechanosensory function (Cloudsley-Thompson 1955; Foelix and Müller-Vorholt 1983; Brownell 1989; Gaffin and Brownell 1997; Wolf 2008, 2017; Knowlton and Gaffin 2011). Intriguingly, the pectines are located on the ventral side of the second mesosomal segment, just behind the walking legs and the genital operculum. Most studies suggest that pectines function as a substrate/contact chemosensory organ which is involved in mate localization and, with lesser evidence, in prey trailing/localization (Krapf 1986; Gaffin and Brownell 1992, 2001; Melville et al. 2003; Steinmetz et al. 2004; Taylor et al. 2012). Recently, it has been proposed that scorpions can retrace their own paths using contact autochemosensation, or recognize chemical gradients in the area surrounding their burrows (Gaffin and Brayfield 2017).

Besides the pectines, scorpions possess chemoreceptive hairs on their tarsal leg segments (Foelix and Schabronath 1983) and pedipalps (Steinmetz et al. 2004). In the latter, a special field of chemosensory sensilla on the chelae of the pedipalps has been identified and termed the constellation array (Fet et al. 2006a, b). Abushama (1964) hypothesized that small hairs, termed trichobothria, distributed over the pedipalps might be responsible for detecting airborne chemicals. Recently, Nisani and colleagues (2018) demonstrated that the scorpion *Paruroctonus marksi* avoids airborne scents derived from a predator. By performing ablation experiments of chemosensory sensilla on the pedipalps, this ability diminished significantly. Scorpions also use their chemical sense to orient toward water, and could potentially locate areas of moist substrate near the burrow entrance (Abushama 1964; Gaffin et al. 1992). Chemosensory hairs on tarsal segments have been identified as the most important structures for the detection of substrate moisture (Gaffin et al. 1992). In addition, the tarsal organs on the dorsal aspect of the tarsal segments are very sensitive to water vapor and may mediate orientation towards higher humidity areas (Gaffin et al. 1992).

#### Vision

Scorpions have a dorsal pair of median eyes and 2–5 lateral eyes (three in *Mesobuthus eupeus*, see Fig. 1) on either side of the anterior carapace (Locket 2001). The median eyes may be able to form low-resolution images, while the lateral eyes are highly sensitive to light but lack an image-forming lens (Locket 2001). The cells in scorpions’ eyes contain shielding pigments which migrate away at night to drastically increase sensitivity to light compared to during the day (Locket 2001). Both sets of eyes are most sensitive to green light (~ 500 nm), with a secondary peak of sensitivity in the lateral eyes to ultraviolet light (350–400 nm), and are insensitive to red or infrared (IR) light (> 675 nm) (Machan 1968; Fleissner and Fleissner 2001b). Based on physiological evidence, scorpions can apparently see 360° around their body (Locket 2001), and the median eyes of some desert scorpions may have sufficient visual acuity and sensitivity to use horizon landmarks for orientation, even on moonless nights (Angermann 1957; Fleissner 1977b; Schliwa and Fleissner 1980). Visual guidance toward the burrow could in theory be mediated by scene familiarity as described by Baddeley et al. (2012). Starlight and moonlight (astromenotaxis) can also guide certain scorpions (Linsenmair 1968). Physiological evidence on the structure of the eyes has led Locket (2001) to speculate that scorpions may be able to use the sky’s polarized pattern of light for orientation and/or navigation, and initial behavioral evidence exists for a response to polarized light (Brownell 2001). The polarized light hypothetically used by scorpions probably originates from the moon, since polarized light directly from the sun is not available 1 h after dusk (Stair and Johnston 1953). Nonetheless, polarized moonlight is sufficient for orientation, as the dung beetle exemplifies (Dacke et al. 2003). Optic flow (the motion of objects in the visual field as an organism moves through space) as a measure of distance traveled has never been observed in scorpions, but as Warrant and Dacke (2010) posit, nocturnal optic flow could be possible as long as the visual features are present and detected. Beyond ocular vision, scorpions also have non-retinal photoreceptors in their tail (Zwicky 1968, 1970a, b; Rao and Rao 1973), and some have hypothesized that they can use their entire cuticle to collect and amplify ultraviolet (UV) light (Gaffin et al. 2012). Along these lines, scorpions may compare light intensities between ocular, metasomal, and cuticular photosensors to ascertain whether a portion of their body is under shelter, and thereby orient toward the relative darkness of the shelter (Gaffin et al. 2012).

Although the resolution of scorpion eyes has been suggested to be rather low (Locket 2001), their extreme sensitivity to light has implications for their behavior. Scorpions are negatively phototactic, meaning they exhibit a directed escape response to a comparatively darker region when illuminated by bright light (Abushama 1964; Torres and Heatwole 1967; Camp and Gaffin 1999; Fleissner and Fleissner 2001a). Scorpions most strongly avoid UV light and green light to which their eyes are most sensitive (Machan 1968; Blass and Gaffin 2008). Exposure to UV and green light disrupts normal locomotion, resulting in positive photokinesis, i.e., movement that is faster and more sporadic than under IR, red, and no light (Blass and Gaffin 2008; Gaffin and Barker 2014).

### Homing behavior in scorpions

In general, our knowledge of shelter choice, shelter/burrow fidelity, and homing behavior in scorpions is very scarce and mostly limited to desert-dwelling species. Due to their harsh habitat, the scorpion’s ability to find shelter drastically increases its chances of survival by providing protection from temperature extremes, desiccation, and predation (Hadley 1974; Polis and Farley 1980; Polis et al. 1986). Thus, the ability to return to an adequate and already known shelter/burrow would be of extreme importance to these animals, especially considering the low number of adequate shelters in these comparatively ecologically simple environments. Indeed, evidence of homing exists. It is not uncommon for *Paruroctonus mesaensis* to be faithful to the same burrow for time spans ranging from months to years (Polis et al. 1986). In an in situ observational study, *Mesobuthus gibbosus* scorpions oriented their movements non-randomly toward a stone wall which provided shelter for some members of the population (Kaltsas and Mylonas 2010). Members of the genus *Paruroctonus* have also been observed returning directly and in straight lines to their burrows in the field (Polis et al. 1986). Additionally, non-random, directional homing behavior of desert scorpions has been observed in the laboratory with artificial shelters (Bost and Gaffin 2004; Vinnedge and Gaffin 2015).

However, a detailed analysis of homing behavior has not been performed, and the question of which sensory mechanisms are involved has not been addressed so far. Scorpions are rather difficult as experimental animals, as their motivation for specific behaviors, including homing, is not easy to recognize. For example, it is known that ants will go straight home when a food item has been collected. So far, features like this are not known to exist in scorpion behavior. Furthermore, most scorpions do not necessarily show high home fidelity to a single shelter. In this sense, establishment of home sites by choice is rather difficult to allow in the laboratory due to time and space limitations. These factors lead to low success rates and hamper straightforward behavioral experiments using scorpions as model organisms. This paper sets out to present an improved setup to investigate and provide the first evidence of homing behavior in the scorpion *Mesobuthus eupeus*. We conduct an analysis that allows discrimination of direct navigation towards the shelter. To test for idiothetic cues, we analyze directed movements in blinded scorpions, thus presenting a pioneering work on path integration in scorpions.

## Materials and methods

### Animals

**Fig. 2:**
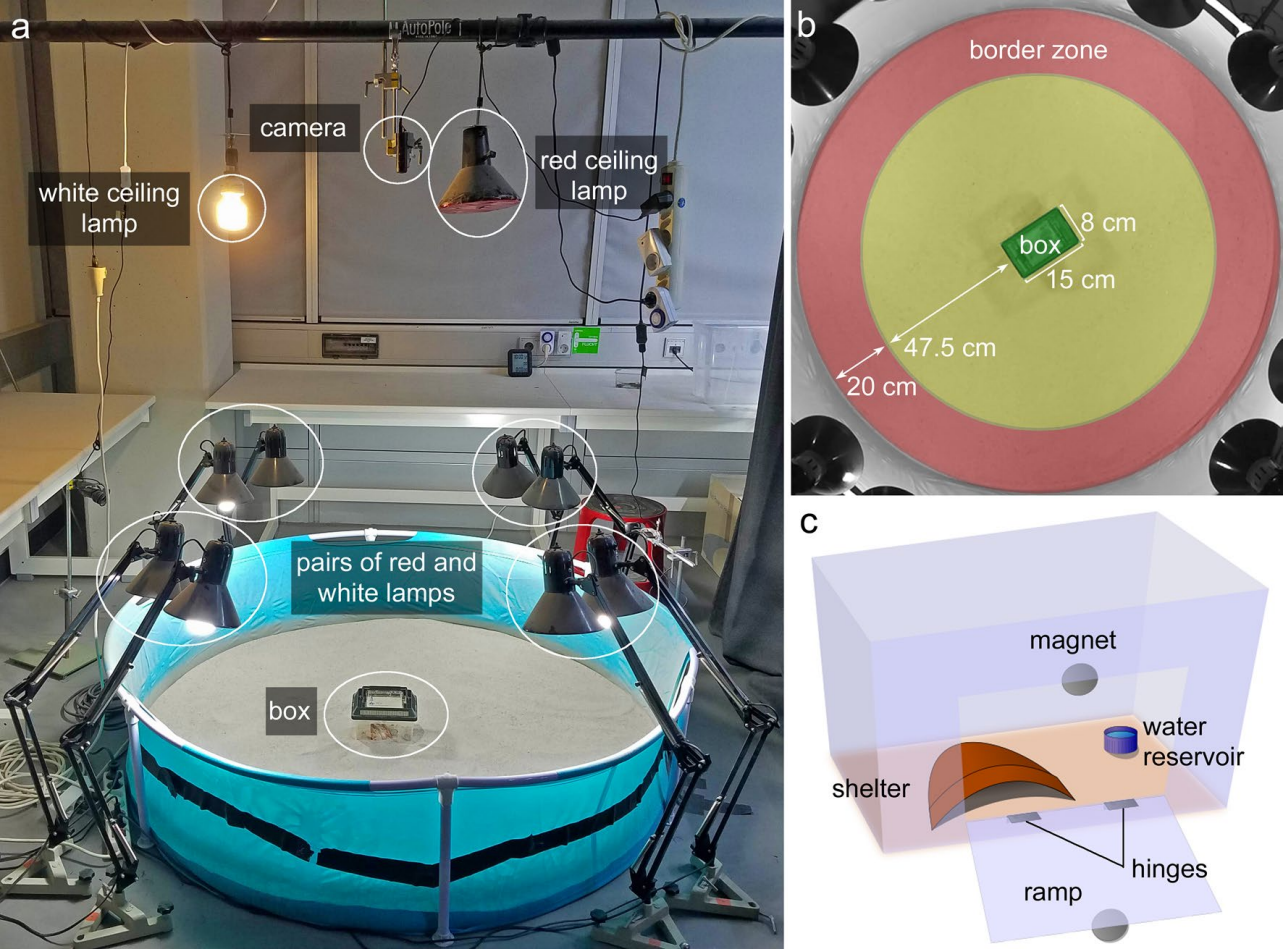
The setup used in the present study. **a** Photo of the arena with labeling of all relevant elements. Four pairs of floor lamps (with one red light and one white light per pair) plus a hanging white light and red light illuminate the arena. A sport camera films the trial from directly above. **b** Photo of the arena from above depicting dimensions of the box and border zone. **c** Example of fauna box (lid omitted for clarity) containing a terracotta shard and water reservoir. A magnet allows the box to be opened during a trial

Forty-eight adult individuals of the lesser Asian scorpion, *Mesobuthus eupeus*, were purchased from the Pet Factory (https://thepetfactory.de/). Scorpions were individually housed in clear plastic fauna boxes measuring 15 × 8 × 12 cm (Fig. 2c). Each box contained a 2–3-cm layer of sand (WECO, Sahara Spielsand), a plastic Falcon tube cap for water, and a shard of curved terracotta pottery for shelter. Scorpions were supplied with water three times weekly and a cricket (*Acheta domesticus*) once every 2 weeks. Three times per week, the inside of the fauna box was sprayed with water to create sand of a consistency conducive to burrowing. All experiments were carried out in the same room that the scorpions were held in, which was kept at 26–30 °C and 47–49% relative air humidity. The boxes each had a 7.5 × 6.75 cm portion of one wall cut out (Fig. 2c). The portion was reattached via hinges at the bottom and a magnetic closure at the top so that it could be opened during experiments to create a ramped exit for the scorpion to leave and re-enter the box at will. Voluntary departure from the box was deemed necessary because of the high failure rate in preliminary trials, probably due to stress from being handled. This also ensured that the animals’ movements in a trial were not due to a panic response to handling. The animals were kept under a 14:10 h light:dark reverse photoperiod with gradual light changes at imposed dusk and dawn. The scorpions had been entrained to this photoperiod for at least 4 months prior to testing. The experimental area was shielded from light in the animal storage area (and vice versa) by heavy, dark curtains.

The dorsal mesosomata of the test animals were painted with a blue acrylic touch-up paint pen (MOTIP DUPLI, Dupli-Color Lackstift No. 120-0100) a minimum of 27 days before testing. In preliminary studies, this color paint was determined to provide the best contrast for computer-aided tracking purposes against the light-colored sand used here. For the best contrast under IR light in the IR trial condition (see below), the mesosomata were instead painted white (MOTIP DUPLI, Dupli-Color Lackstift No. 0-0750) at least 18 days before testing. Previous studies found that painting the mesosomata did not seem to affect the activity of the scorpions (Tourtlotte 1974; Kaltsas and Mylonas 2010).

### Apparatus

The test arena was comprised of a 580-L PVC-polyester Bestway frame pool filled with a 2–4-cm layer of sand (Fig. 2a). The pool measured 150 cm in diameter and 38 cm in height. Evenly spaced on the floor around the outer perimeter of the arena were four pairs of spring-balanced lamps (Fig. 2a). Lamps in each pair were situated directly next to one another, and fitted with an Exo Terra 25 W natural light full-spectrum daylight fluorescent bulb and an Osram 1.6 W red LED bulb, respectively. The red floor lamps emitted light wavelengths from ~ 430 to ~ 760 nm, with the main spectral peak at ~ 624 nm (OceanOptics Red Tide USB650 spectrometer; OceanView software version 1.6.7). There were two secondary peaks about 4% the intensity of the main peak centered at ~ 515 nm and ~ 455 nm. The bulbs of the floor lamps were about 55 cm above the surface of the arena (elevation approximately 55° from the center of the arena). A Manfrotto Autopole tension bar was positioned across the entire room above the arena. From it, two lamps were hung (Fig. 2a)—one fitted with a white 25 W Exo Terra Reptile UVB100 fluorescent bulb and one with a Toshiba 60 W warm white LED bulb equipped with red cellophane covering the opening of the lampshade (elevation of both lamps approximately 75° from the center of the arena). The hanging red lamp produced light wavelengths from ~ 440 to ~ 760 nm, with the main spectral peak at ~ 610 nm and a secondary peak at ~ 540 nm which was about 9% the intensity of the main peak. Another small peak was measured at ~ 450 nm at less than 1% the intensity of the main peak. This lighting setup was chosen to eliminate shadows in the arena, as well as to brightly and evenly illuminate the arena in both white light and red light scenarios. The white lamps provided a bright-light condition of 630 lx (PCE-EM 882 environmental meter) at the arena substrate, which was presumed to be a noxious stimulus. The red lamps provided a low-light condition of 65 lx to simulate nighttime while still allowing video tracking.

All lamps were operated via a remote control synchronized to SilverCrest radio outlet attachments. This allowed the light conditions to be manipulated without entering the room and potentially disturbing the test animal. Two webcams (Microsoft, LifeCam HD-3000; Logitech, HD Webcam C270) were positioned on ring stands on opposite sides of the arena, externally. They were connected to a laptop located outside the room so that the scorpion’s activity could be remotely monitored during a trial. The arena was recorded by a Sony HDR-AS50 sport camera positioned on the tension bar 145 cm above the center of the arena (Fig. 2a).

Based on ambiguous results obtained from this setup (see below), we replaced the red overhead lamp and red floor lamps with two overhead IR spotlights (RayTEC Var2-i2-1, wavelength 850 nm, elevation approximately 75° from the center of the arena) in the IR trial condition. Consequently, the sport camera was exchanged for a Basler acA1300-60gm camera equipped with an IR manual iris lens (computar Varifocal H3Z4512CS-IR). Videos were recorded using the software Media Recorder 4.5 (Noldus).

### Trial procedure

Before a trial, the sand in the arena was turned over with a garden trowel to ensure that any chemical trails or footprints from previous trials were disrupted, although the in-floor heaters may have also served to destroy chemical deposits. Then, the sand was smoothed with the trowel to make the substrate as level as possible. Since some scorpions remain in an inactive, quiescent state in their burrows for long periods of time (Polis and Farley 1980; Williams 1987), animals which were visible in their box—rather than hidden under the shelter—were preferentially chosen for trials. During the animals’ imposed night phase, the test subject’s fauna box was placed in the center of the arena in a random orientation with the ramp closed. The orientation was randomized to prevent biases due to inherent and unforeseen cues in the setup, e.g., sand level or lights from equipment LEDs. As the only substantial shelter object in the otherwise exposed arena, the fauna box was intended to be a motivating stimulus for homing behavior. Preliminary trials showed that scorpions spend most of their time at the perimeter of the test arena. Therefore, the likelihood that the scorpion would come across the box by chance was minimized by placing it as far away as possible from the perimeter. After placement, the scorpion was acclimated under red or IR light conditions for 30 min, after which the ramp was opened as carefully and quietly as possible and covered with some sand from the arena to make the substrate in the fauna box roughly flush with the arena substrate. The video camera was then turned on, marking the beginning of phase 1 of the trial. Within the dark control, circadian control, blind, and IR conditions (see below, “Light stimulus experiment procedure” and “Vision experiment procedure” sections), all trials began at the same time—half an hour after imposed dusk for the dark control, blind, and IR trials, and 3 h before imposed dawn for the circadian control trials. The stimulus trials were not controlled in this way to allow multiple trials in a single day, but all analyzed homing bouts except one began within the first hour of the night phase after acclimation. The success rate (the ratio of legitimate to illegitimate trials; see the next paragraph for definition of legitimacy) appeared to decrease as the night phase progressed (data not shown), although the start time did not seem to influence path characteristics of homing bouts.

In all trial conditions except the circadian control condition of the light stimulus experiment (see below, “Light stimulus experiment procedure” section), the test arena was monitored via webcam during phase 1 approximately once every 30 min to see whether the scorpion had left its box. If the scorpion had left the box and was observed walking at the perimeter of the arena, the trial was considered legitimate and phase 2 began. Phase 2 varied by trial condition (see below, “Light stimulus experiment procedure” and “Vision experiment procedure” sections). The perimeter-walking criterion was used so that (1) the length of the straightest return path was roughly equal for all scorpions, and (2) the distance from the scorpion to the nearest edge of the box was at a maximum (67.5 cm), thereby creating the greatest navigational challenge allowed in this setup. Trials were stopped 3 h after the beginning of phase 2 and the animal was removed from the arena. This 3-h time limit was based on preliminary trials in which homing scorpions did so within an average of 103 min after phase 2 began (*n* = 4, SE = 44.67). Trials were considered illegitimate if a scorpion was not observed at the arena perimeter within 3 h of phase 1. This was based on preliminary trials which showed that scorpions who left their boxes did so within an average of 71 min (*n* = 11, SE = 12.96).

Scorpions were given multiple opportunities (maximum six trials) to perform a homing bout, but 31 out of 40 bouts occurred in the first trial of an experimental condition. Individuals were tested with a minimum of 2 days’ rest in between. The same animals were tested in all trial conditions of the light stimulus experiment and the blind trials, but 20 new scorpions were used for the IR trial condition to avoid the effects of the eye paint (see below). Six scorpions performed homing bouts in more than one trial condition: one individual homed in all four conditions, one individual in three, and four individuals in two conditions. Repeated homing bouts occurred with at least 9 days in between (*M* = 76 days, maximum = 163 days). A repeated-measures design was not implemented in statistical analysis because the same scorpions did not perform a homing bout in all conditions.

#### Light stimulus experiment procedure

In the first experiment, we examined whether a sudden and unexpected light stimulus during the imposed night phase would affect the likelihood or characteristics of homing behavior. Three trial conditions were specified: stimulus, dark control, and circadian control. In the stimulus trials, the white lights were remotely switched on and the red lights were switched off when the perimeter-walking criterion had been met, marking the beginning of phase 2. The dark control trial procedure was the same, except the time was simply noted at the beginning of phase 2 and the red lights were kept on. To determine whether the sudden illumination itself had an effect or whether illumination must be unexpected to influence homing behavior, the white lights were turned on in the circadian control condition at the regularly scheduled time of imposed dawn to which the scorpions had been acclimated. In other words, the same illumination as in the stimulus condition was applied, but at the expected time of entrained dawn. To accomplish this, phase 1 of the trials began 3 h before imposed dawn. At dawn, phase 2 began by automatically turning off the red lights and turning on the white lights via 24-h electromechanical timer switches. In the light stimulus experiment, only phase 2 homing bouts of legitimate trials were analyzed to ensure that the correct lighting and timing conditions were applied, regardless of whether it was the first bout in the trial.

#### Vision experiment procedure

The results of the light stimulus experiment (see below) suggested that homing bouts performed under red light may be less directionally consistent than those performed under white light. Based on this, we designed a second experiment to determine (1) whether ocular vision is necessary for homing, and (2) whether observed differences in homing behavior under white light and red light were due to reduced visual capacity or due to a reaction to ambient light conditions per se. To this end, three trial conditions were created: sighted white light (animals tested under white light with eyes intact), sighted red light (under red light with eyes intact), and blind white light (under white light with eyes covered). Homing bouts from trials in the light stimulus experiment were re-categorized based on whether the homing bout occurred under red light or white light. We ruled that homing bouts in phase 2 of the stimulus, circadian control, and IR trial conditions—i.e., under white light—could be combined because no significant differences were found between them in terms of path characteristics (see “Results” and Supplementary Tables). Furthermore, only the first homing bout in a trial was analyzed in the vision experiment, regardless of the phase in which it occurred and the overall trial legitimacy according to the criteria of the light stimulus experiment. This was to eliminate any potential learning effects. It should be noted that illegitimate trials occasionally produced homing bouts which met the definition stated in “Analytical methods” below, and also that all analyzed homing bouts began at the same distance from the box. Scorpions in the blind condition were tested after painting the median and lateral eyes with two coats of opaque dark blue acrylic touch-up paint (MOTIP DUPLI, Dupli-Color paint stick No. 20-0804). Blind trials were conducted a minimum of 24 h after painting. After a successful trial, the subject’s eyes were inspected under a light microscope to verify that they were still covered with paint. The blind trials followed the same procedure as the trials in the stimulus condition of the light stimulus experiment, such that the white lights were turned on at the beginning of phase 2. To investigate ambiguous results comparing the behavior of blind and sighted animals under red light conditions, an IR setup was used (see “Apparatus” section). The procedure was the same as in the stimulus condition explained above (see ‘Light stimulus experiment procedure’), but the red light in acclimation and phase 1 was simply replaced with IR light.

### Analytical methods

Footage of each trial was analyzed with the tracking program EthoVision XT version 13.0 (Noldus). The origin of the tracking program’s coordinate plane was centered at the center of the arena. The dynamic subtraction method of detection was used, which detects the animal by subtracting the current frame from a constantly updating background image. Video of the scorpion’s movements was then tracked at a sampling frequency of 0.5 samples/s. The period of time between sampling points will hereafter be referred to as a step. The trial time, *x*- and *y*-coordinates of the scorpion’s position, distance traveled from the last sample point, and instantaneous velocity were given for each sampling point in the raw data exported from EthoVision. After obtaining the raw data, Microsoft Excel and the statistics software R version 3.5.2 and R Commander version 2.5-1 were used to analyze the incidence and path characteristics of the homing bouts (see below).

In EthoVision, a line of determination was defined to identify the beginning of a homing bout. This imaginary line was set 20 cm from the inner wall of the arena (Fig. 2b). The margin between the wall and the line of determination was termed the border zone, the minimum width of which was defined according to the average size of the species: the border zone should be at least wide enough that the animal can walk inside it without direct physical contact to either margin. The maximum width was defined by observing preliminary trials and subjectively deciding whether or not a scorpion’s locomotion appeared consistent within that margin. In other words, scorpions exploring the edge of the arena did so generally within a margin of 20 cm, and traversals of the arena appeared to be distinct patterns of movement. Therefore, crossing this line of determination was deemed a deliberate departure from the border zone. A return journey, or homing bout, was defined as the sum of all sampling steps from the last point in the border zone to the first point at the edge of the box, or inside the box if available. Similarly, an outbound journey, or departure bout, was defined as all steps from the last point in the box zone to the first point across the line of determination in the border zone. In comparison to the homing bout, the home vector (HV) was defined as the shortest path between the last position in the border zone and the center of the box, as determined by EthoVision.

To reduce noise, steps during which the scorpion was not moving were excised from the homing and departure bouts. These pauses were defined as a period during which a scorpion’s instantaneous velocity was slower than 0.333 cm/s for at least 2 s—the length of one sampling step in the tracking program. This threshold was determined by comparing preliminary footage of walking scorpions to the instantaneous velocity measurements at the corresponding sampling points. At the points when it was subjectively decided that the scorpion was not walking, the instantaneous velocity was under 0.333 cm/s. Instantaneous velocity was not registered by the tracking program as 0 cm/s if the scorpion shifted slightly or turned in place, or even if the scorpion was at a complete standstill, because the apparent center point of the animal would move slightly with each sample according to the program. A subjective determination of movement was therefore necessary to remove this noise.

#### Trial success and homing rate analysis

**Fig. 3:**
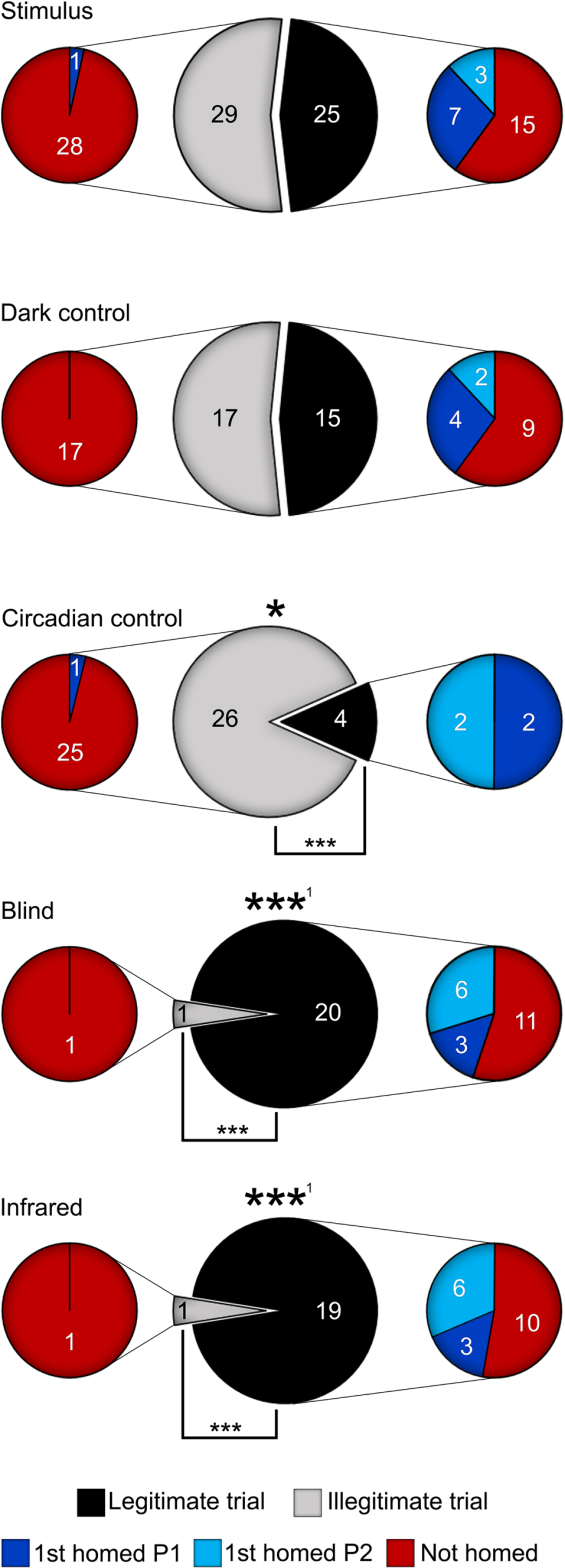
Number and success rates of trials and frequency of homing bouts in the different conditions (stimulus, dark control, circadian control, blind, and infrared) separated into illegitimate and legitimate trials (see “Materials and methods” “Trial procedure” for definitions). Homing events are separated by the phase (P1 or P2) in which the first homing bout occurred. Significantly different distribution of a trial condition compared to all conditions indicated by *p < 0.05 and ***p < 0.001. ^1^Denotes that conditions differed from other conditions but did not differ from each other; significance between categories within a condition indicated by ***p < 0.001 (Pearson’s chi-squared multiple comparison tests with FDR *p*-value adjustments)

Since scorpion behavioral research is generally hampered by low participation rates, aspects of the experimental setup and procedure which influenced the efficiency of the study were examined. The effect of the trial condition itself on the total trial success rate was analyzed by comparing counts of legitimate and illegitimate trials in the different trial conditions with a Pearson’s chi-squared test (*n* = 157) and post hoc multiple comparison tests with FDR *p*-value adjustments. All attempted trials were analyzed according to their original trial condition: stimulus (*n* = 54), dark control (*n* = 32), or circadian control (*n* = 30) from the light stimulus experiment, and blind (*n* = 21) or IR (*n* = 20) from the vision experiment (Fig. 3).

To determine whether the trial condition influenced the frequency with which the scorpions exhibited homing behavior, a Fisher’s exact test (*n* = 83) was used to analyze the incidence of homing versus non-homing in all legitimate trials according to the five original trial conditions (stimulus, dark control, circadian control, blind, or IR).

#### Departure-homing comparison

**Fig. 4:**
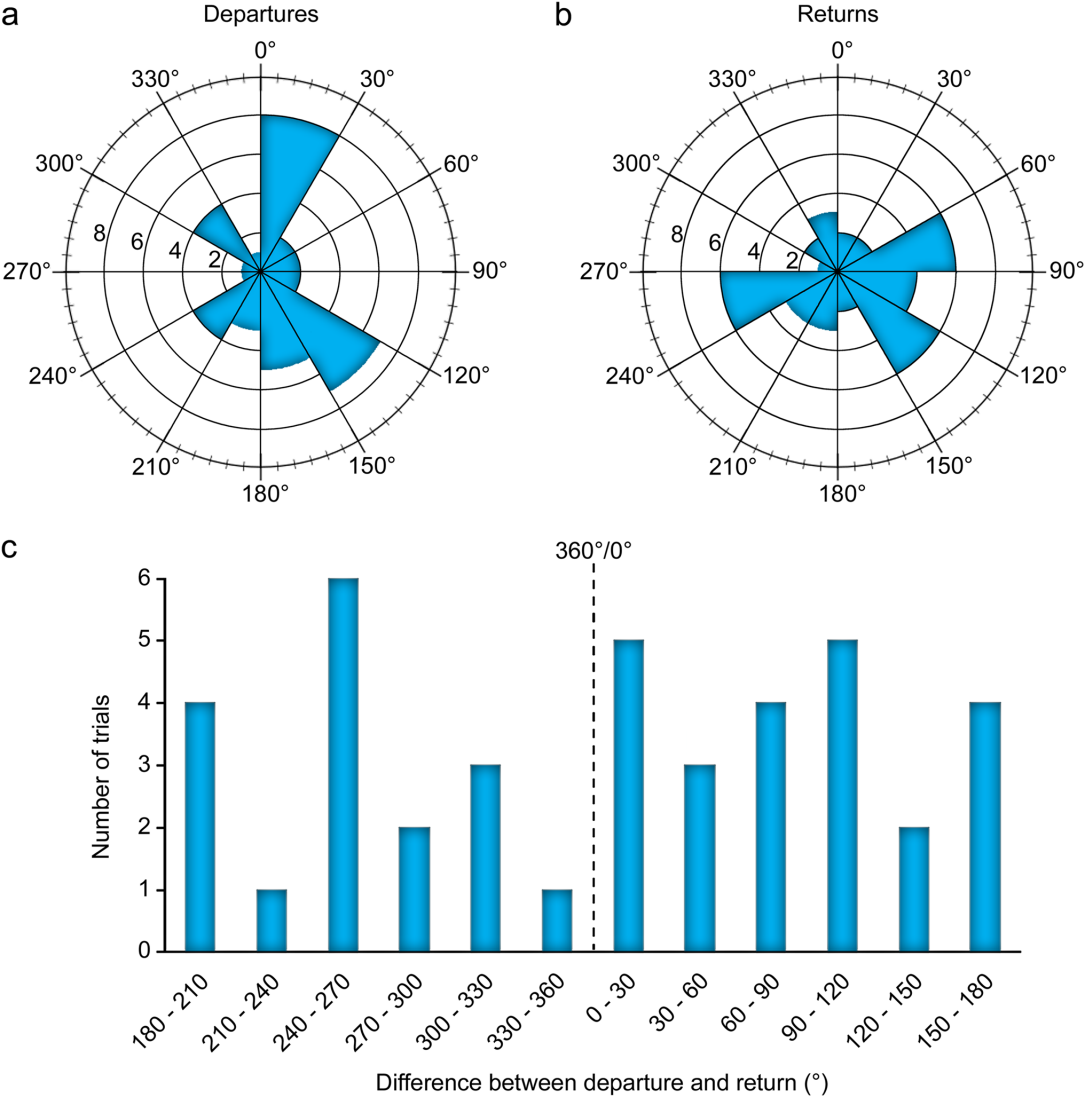
Angular comparisons of departures and returns. **a** Angles of departures depicted in 30° steps. **b** Angles of returns depicted in 30° steps. Departure and return angles were not significantly different from a random distribution (Rayleigh test and Watson’s test). **c** Angular differences (positive ° clockwise) between departures and returns in 30° steps, indicating that scorpions did not follow their departure path when returning to shelter (Rayleigh test with specified mean direction of 0° and Watson’s test). Dashed line represents 360°/0°

Overall biases for the direction of departure and return were investigated. All first homing bouts were investigated here regardless of trial legitimacy, along with the corresponding departures from the box. The angles of departure and return were calculated in degrees from the sampling point just after or before crossing the line of determination, respectively (Fig. 4). The “top” of the arena’s coordinate plane according to the static orientation of the arena and camera always corresponded to 0°/360°. To see whether the distributions of departure or return angles were randomly distributed, a Rayleigh test of circular uniformity (*n* = 40) was performed on the angles. Since a bimodal distribution of departure angles was suspected from visual inspection of the data, a Watson’s test was also applied to find multimodal violations of circular uniformity (Ruxton 2017).

The similarity of each animal’s outbound and return journeys was compared by calculating the difference from the departure angle to the return angle (in positive ° clockwise). If homing bouts were oriented preferentially toward the same direction as the angle of departure, one would expect a unimodal bias toward 0°/360° of difference. The angular differences were analyzed for directional bias with a Rayleigh test of circular uniformity with a specified mean direction of 0° (*n* = 40). Multimodal biases were investigated with a Watson’s test of circular uniformity. To confirm that the homing bouts did not match outbound paths, a visual comparison of the departure and return bouts was also performed.

#### Directional deviation analysis

**Fig. 5:**
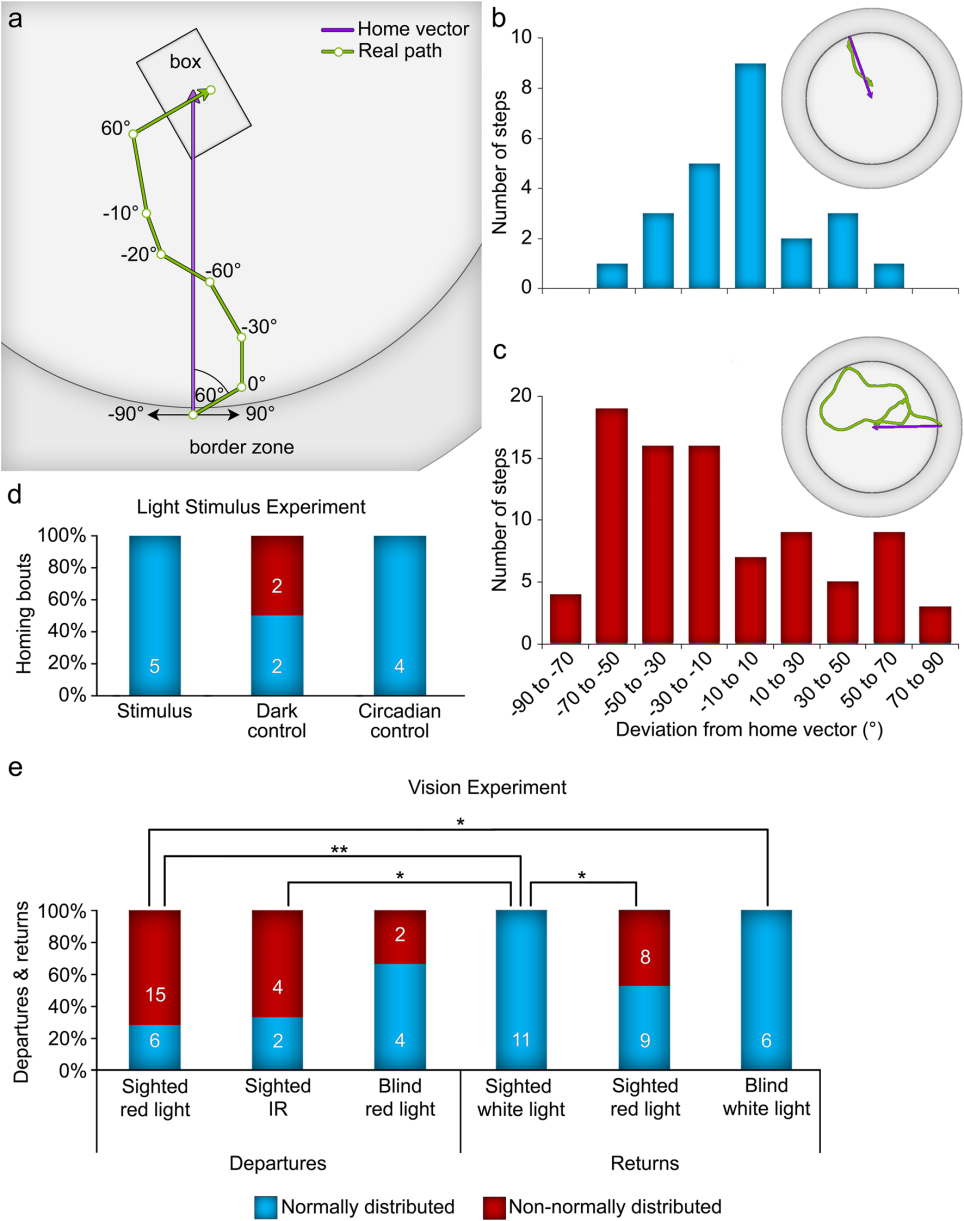
Analysis of the directional deviation from the home vector. **a** Example of a homing bout to show the measurement of angles (for further information on calculations, see “Materials and methods”). **b** Example of a homing bout in which deviations from the home vector were normally distributed. **c** Example of a homing bout in which deviations from the home vector were non-normally distributed. **d** Frequency of normality in directional deviation for legitimate homing bouts in the three conditions of the light stimulus experiment. **e** Representation of normality in directional deviation for the departures and first homing bouts per trial in the vision experiment. Significance between conditions indicated by *p < 0.05 and **p < 0.01 (Fisher’s exact multiple comparison tests with FDR *p*-value adjustments)

To analyze the directional adherence of a homing bout to the HV, the compass direction of each step was computed and transformed to represent a deviation (in °) from the compass direction of the HV, which was transformed to 0° (Fig. 5). From the perspective of one at the beginning of the HV looking toward the end, a step whose directional trajectory deviated to the left of the HV’s direction was negative, while a directional deviation to the right was positive. The deviations were also transformed to ignore forward and backward directionality, and thereby accounted for potential overshooting of the box and subsequent backtracking which would be ~ 180° deviated from the HV. If a scorpion followed the direction of the HV overall, the distribution of deviations would be statistically normal around 0°. The deviations of a scorpion’s return journey were, therefore, analyzed with the Shapiro–Wilk and Kolmogorov–Smirnov tests of normality. Two tests were used to ensure the legitimacy of the normality assumption, since some of the sample sizes for steps were very small (range: 5–285 steps) and could result in erroneous rejection or confirmation of the normality assumption. A set of deviations was considered normal only if both tests upheld the assumption of normality. Finally, the counts of normally and non-normally distributed deviations—i.e., the number of scorpions who were directionally consistent with the HV versus those who were not—were analyzed separately in the light stimulus experiment (*n* = 13) and the vision experiment (*n* = 34) by trial condition. Furthermore, the frequencies were counted for the departure bouts (*n* = 33) according to whether the animals were sighted under red light, sighted under IR light, or blind under red light. Owing to expected frequencies smaller than five, a Fisher’s exact test and post hoc multiple comparison tests with FDR *p*-value adjustments were used in each analysis to determine whether the observed frequencies differed significantly from each other.

#### Lateral displacement analysis

**Fig. 6:**
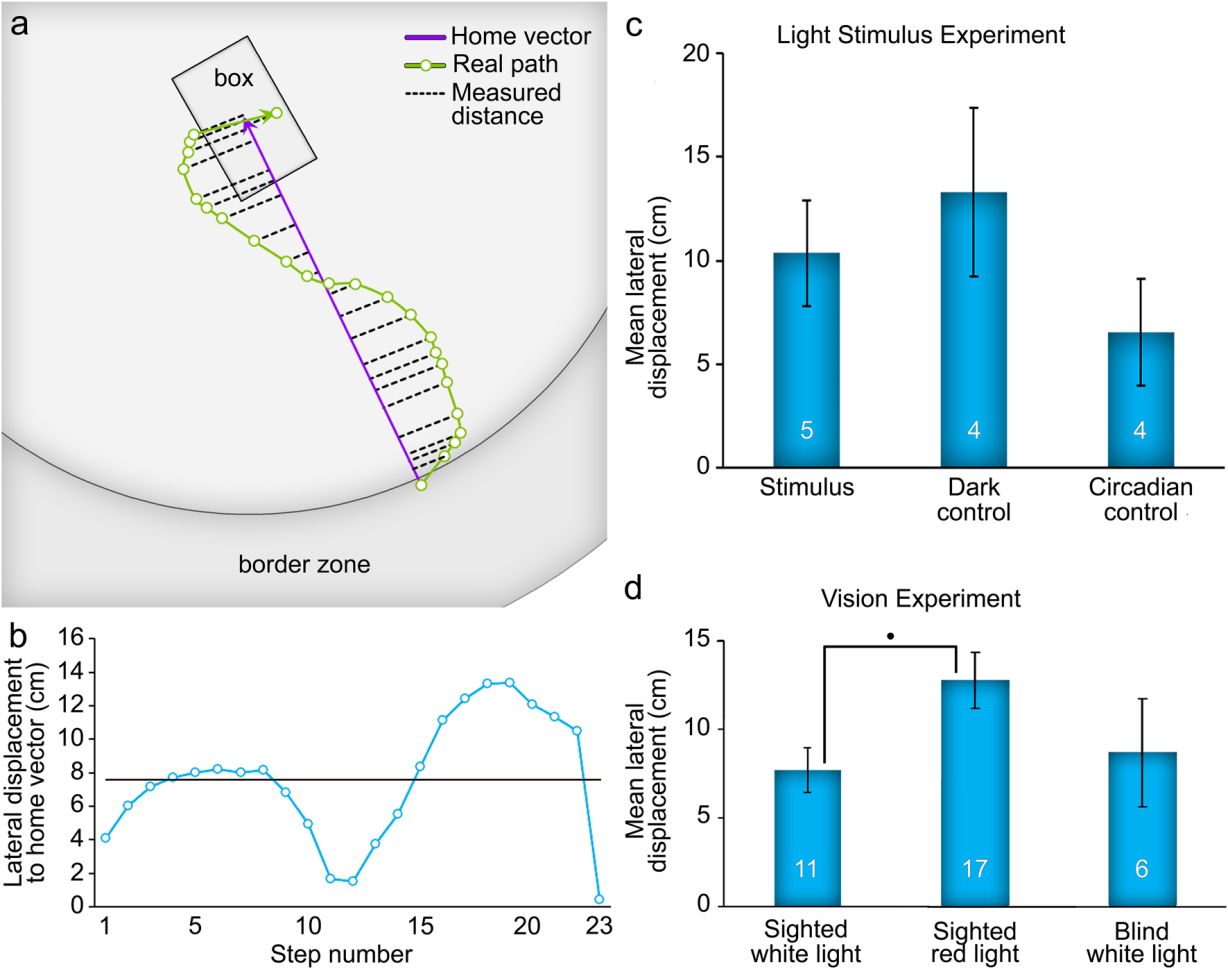
Analysis of the lateral displacement from the home vector. **a** Example of a homing bout to show the measurements of orthogonal distance to the nearest point on the home vector (for further information on calculations, see “Materials and methods”). **b** The values of lateral displacement for each step (in cm) of the homing bout depicted in a. Black line represents the mean (7.58 cm). **c** Mean ± standard error of lateral displacement for legitimate homing bouts in the three conditions of the light stimulus experiment. **d** Mean ± standard error of lateral displacement for the first homing bouts per trial in the three conditions of the vision experiment. Significance between conditions indicated by •*p* < 0.1 (ANOVA post hoc Tukey test)

A parameter was designed to measure how closely the animal’s position matched that of the straightest homeward trajectory (Fig. 6). This provided information on the precision of a homing bout regardless of length, which could be inflated if the bout was tortuous yet centered closely on the HV. The scorpion’s perpendicular distance to the nearest point on the HV was calculated at each sampling point, and subsequently averaged across all sampling points to give a so-called lateral displacement value for each homing bout. A greater average lateral displacement from the HV indicated a less precise positional adherence to the HV. The mean lateral displacement and the standard error of the mean were calculated for each trial condition, separately and respectively for the light stimulus experiment (*n* = 13) and the vision experiment (*n* = 34). An ANOVA was used to determine whether the lateral displacement of homing bouts was affected by trial condition in each experiment.

#### Distance efficiency analysis

**Fig. 7:**
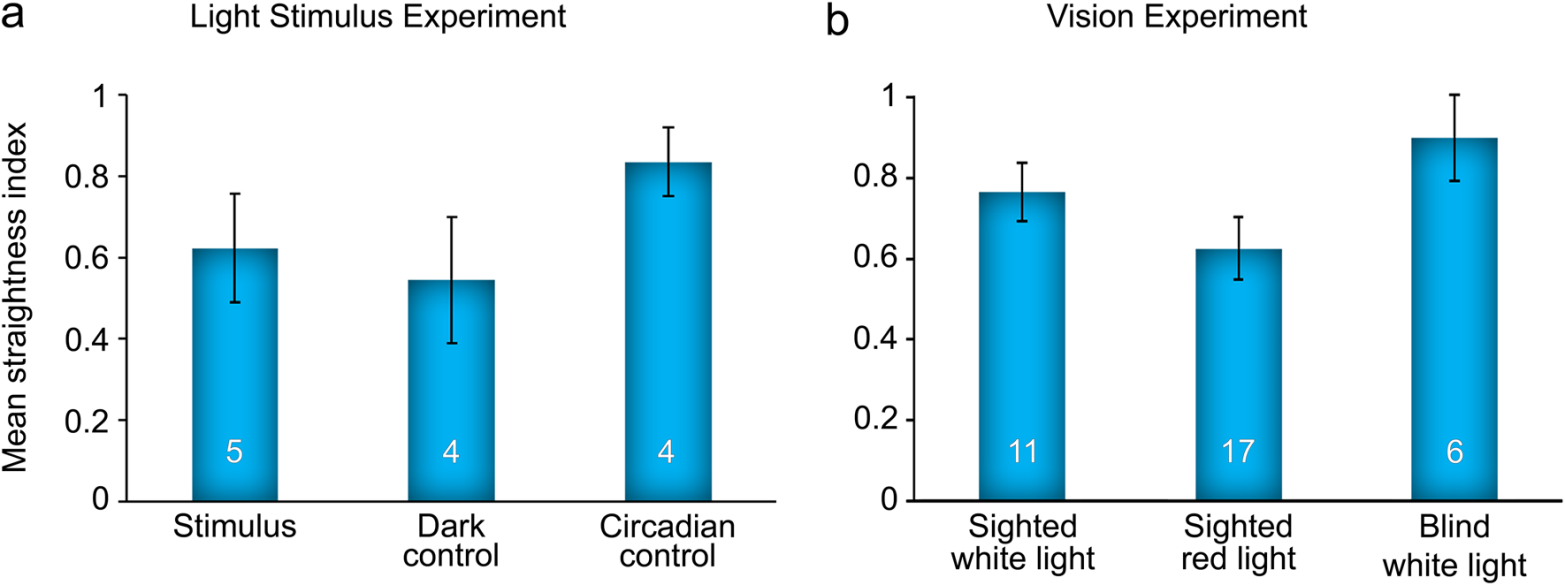
Analysis of the straightness indices. **a** Mean ± standard error of straightness indices for legitimate homing bouts in the three conditions of the light stimulus experiment. **b** Mean ± standard error of straightness indices for the first homing bouts per trial in the three conditions of the vision experiment

To measure the efficiency of a homing bout relative to the shortest path, the length of the HV was divided by the length of the actual return path (Fig. 7). This parameter was termed the straightness index (SI). A perfectly direct return journey would have an SI of 1.00, while a meandering or tortuous return would have an SI less than 1.00. Mean SI and standard error were calculated for each trial condition. An ANOVA was applied to determine whether the SI of homing bouts was affected by trial condition within the light stimulus experiment (*n* = 13) and vision experiment (*n* = 34), separately.

All analyzed trials are depicted in the electronic supplement (Fig. S1). Furthermore, all parameters of the analyzed trials, observed and expected frequency counts, and post hoc comparisons of significant and near-significant statistical tests are accessible in the Supplementary Tables.

## Results

### Trial success and homing rate

In the stimulus trials of the light stimulus experiment involving sighted scorpions, 11 individuals out of 54 total trials (20%) returned to their box (Fig. 3), five of which resulted in a legitimate phase 2 homing bout according to the light stimulus experiment trial legitimacy criteria (scorpions having been observed at the arena perimeter within 3 h of phase 1). Eight of the 11 homing scorpions performed their first homing bout under red light in phase 1—one of which occurred in a trial scored as illegitimate—while three first homed under white light in phase 2. The remainder of the trials did not result in a homing bout. Six out of 32 (19%) sighted scorpions returned in the dark control trial condition, all of which were by definition under red light. Four trials contained legitimate phase 2 homing bouts which were analyzed in the light stimulus experiment, but the first-occurring homing bout was in phase 2 for only two trials. The other four trials had homing bouts that first occurred in phase 1. In comparison, 5 out of 30 (17%) scorpions returned in the circadian control condition. Three animals performed a legitimate phase 2 homing bout which was analyzed in the light stimulus experiment, but only two of these were the first-occurring homing bout of the trial. Two homing bouts first happened in phase 1 under red light. The fifth homing bout was from a trial that did not meet legitimacy—the scorpion departed and homed outside the allotted time of the experiment after phase 2 under white light. Nine out of 21 (43%) blind trials resulted in a homing bout, all of which occurred in a legitimate trial. Six of the homing bouts occurred during phase 2, and were consequently under the white light condition. The other three bouts occurred in phase 1 under red light. Nine out of 20 (45%) IR trials contained homing bouts. Three scorpions first homed in phase 1, and six first homed in phase 2, all in legitimate trials. Unless otherwise stated, the phase 1 homing bouts of blind and IR trials will be excluded from the following analyses because there were too few (*n* = 3 for both conditions) to accurately analyze.

Pearson’s chi-squared test found that the overall frequency distributions of legitimate and illegitimate trials in the stimulus, dark control, circadian control, blind, and IR trial conditions differed significantly from expected at the 95% confidence interval, χ^2^(4, *n* = 157) = 49.592, *p* < 0.001 (see Supplementary Tables for observed and expected values, and all pairwise comparisons). Post hoc tests revealed that trials in the circadian control condition were less likely to succeed than all other conditions, while blind and IR trials were the most likely to succeed, and equally so compared to one another (*p* = 0.91). Furthermore, the number of legitimate and illegitimate trials within a trial condition differed significantly from each other in the circadian control, blind, and IR trials, such that circadian control trials were more likely to fail than to succeed (*p* < 0.001), and conversely blind trials (*p* < 0.001) and IR trials (*p* < 0.001) were more likely to succeed than to fail. Proportions of legitimate and illegitimate trials within the stimulus and dark control conditions did not differ, nor did the legitimacy rates differ between these two trial conditions.

Fisher’s exact test determined that the frequencies of homing and non-homing legitimate trials did not differ significantly from expected at the 95% confidence interval, *n* = 83, *p* = 0.29.

#### Departure-homing comparison

Directional biases were examined in the angles (in °) of the initial departure and return according to the point after or before, respectively, the animal crossed the line of determination. All trials involving a homing bout were analyzed here (see Fig. 4a, b). A Rayleigh test of circular uniformity found that departure angles did not display a unimodal violation of random distribution, *z*(*n* = 40) = 0.114, *p* = 0.59. Since a bimodal distribution was suspected from visual inspection of the data (Fig. 4a), specifically in the categories of 0° to 30° and 120° to 150°, a Watson’s test for circular uniformity was also applied. It did not find a significant departure from circular uniformity at the 95% confidence interval, *U*^2^(*n* = 40) = 0.0676, *p* > 0.10. Return angles were also analyzed (Fig. 4b). Most occurred between 60° and 90°, 120° and 150°, or 240° and 270°, but a Rayleigh test found no significant unimodal violation of random distribution, *z*(*n* = 40) = 0.169, *p* = 0.32, nor did a Watson’s test find significant multimodal violations, *U*^2^(*n* = 40) = 0.0853, *p* > 0.10.

To see whether scorpions followed the same return path back to the box as their outbound path, the angular difference (in °) between the angles of departure and return was calculated (see Fig. 4c). A Rayleigh test of circular uniformity with a specified mean direction of 0° determined that there was no bias toward a mean difference of 0° between departure and return angles, *z*(*n* = 40) = − 0.0483, *p* = 0.67. Multimodal violations of circular uniformity were examined with a Watson’s test, but found no significant violations at the 95% confidence interval, *U*^2^(*n* = 40) = 0.0331, *p* > 0.10. To confirm that the homing bouts did not match outbound paths, the departure and return bouts were visually compared. All departures and returns were determined to be reasonably different (see Supplementary Fig. S1 for all departure and homing bouts).

### Light stimulus experiment

Path characteristics of legitimate phase 2 homing bouts were examined in the stimulus (*n* = 5), dark control (*n* = 4), and circadian control (*n* = 4) trial conditions. An illegitimate circadian control trial which occurred after the allotted time for the experiment is included here, since the homing bout was nonetheless performed under the correct lighting condition.

#### Directional deviation

To compare the adherence to the direction of the HV across trial conditions, the directional deviation from the HV of each step in a return journey was computed (Fig. 5a). Deviations were transformed so that the direction of the HV was at 0°, and each bout’s set of deviations was individually tested for normality. See Fig. 5b and c for examples of homing bouts with normally distributed and non-normally distributed deviations. All five homing scorpions in the stimulus trials as well as all four phase 2 homing bouts in the circadian control trials had normally distributed sets of deviations, whereas only two out of four (50%) in the dark control trials showed a normal distribution (Fig. 5d). A Fisher’s exact test (*n* = 13) was performed to determine whether there was a significant difference in the frequency counts of homing bouts with normally and non-normally distributed step deviations in the three trial conditions. It found that the difference between expected and observed frequencies was not significant, *p* = 0.15.

#### Lateral displacement

The average distance (in cm) that a homing bout strayed to either side of the position of the HV was calculated and averaged within trial conditions (Fig. 6c). See Fig. 6a and b for an example of lateral displacement calculated at each sample point of a homing bout. The dark control condition yielded the greatest lateral displacement (*M* = 13.29, SE = 4.08), while the circadian control condition yielded the least (*M* = 6.54, SE = 2.58). Lateral displacement in the stimulus condition was intermediate (*M* = 10.35, SE = 2.57). An ANOVA (*n* = 13) revealed no significant differences between trial conditions, *F*(2, 10) = 1.11, *p* = 0.37. On average, scorpions strayed 10.08 cm (SE = 1.80) to either side of the HV.

#### Distance efficiency

To examine the effect of trial condition on the distance efficiency of homing bouts relative to the shortest path, the SIs of initial homing bouts were computed by dividing the length of the HV by the length of the homing bout. The mean SIs were compared across trial conditions (Fig. 7a). Homing bouts in the circadian control condition had the highest SI (*M* = 0.83, SE = 0.08), and those in the dark control condition had the lowest (*M* = 0.54, SE = 0.15). The stimulus condition yielded an intermediate mean SI (*M* = 0.62, SE = 0.13). An ANOVA (*n* = 13) determined that these differences were not significant, *F*(2, 10) = 1.26, *p* = 0.32. The overall mean SI was 0.66 (SE = 0.08).

### Vision experiment

Path characteristics of first-occurring homing bouts were compared across sighted white light (*n* = 11), sighted red light (*n* = 17), and blind (*n* = 6) trial conditions to elucidate the effects of lighting and vision. All blind homing bouts analyzed here occurred under bright white light. Three phase 2 bouts previously categorized in the stimulus condition of the light stimulus experiment, two bouts from the circadian control condition, and six bouts from the IR condition made up the newly categorized sighted white light condition in this experiment. Two previously labeled stimulus trials, two dark control trials, and three circadian control trials actually showed their first homing bout in phase 1. These initial homing bouts made up the new sighted red light condition, along with the two dark control trials with their first bout in phase 2, and eight other phase 1 homing bouts from previously unanalyzed trials with no phase 2 homing bouts.

#### Directional deviation

For homing scorpions with sight intact, all 11 under white light showed normal distributions of step deviations, compared with only 8 out of 17 (47%) under the red light condition (see Fig. 5e). All six blinded scorpions had normally distributed deviations in their homing bouts. Counts were also tallied for departure bouts. Only one departure occurred under white light, so it is here excluded. Six out of 21 (29%) departures in sighted scorpions under red light showed normally distributed deviations, compared with two out of six (0.33%) departures in sighted scorpions under IR light and four out of six (67%) departures in blind scorpions. These homing and departure bouts were compared with Fisher’s exact test (*n* = 34 trials, 67 bouts), which found that the observed frequencies differed significantly from expected values at the 95% confidence interval, *p* < 0.001. Post hoc tests with an FDR *p*-value adjustment revealed that a significantly larger proportion of homing bouts was non-normally distributed for the sighted scorpions under red light compared to sighted scorpions under white light (*p* = 0.036), but the differences compared to blind scorpions under white light did not reach significance (*p* = 0.15). There was no difference between returns in the sighted white and blind conditions (*p* = 1.0). In departures, bouts with sighted scorpions under red light were not different from departures under IR light (*p* = 1.0). Frequency counts of departures in blind scorpions did not differ from sighted scorpions under either red light (*p* = 0.29) or IR light (*p* = 0.76), nor were blind departures more or less likely to be normally distributed around the direct vector than any homing conditions. Sighted departures under red light, on the other hand, were significantly less likely to be normally distributed around the direct vector than homing bouts in both the sighted white (*p* = 0.0016) and blind (*p* = 0.023) return conditions. Likewise, sighted departures under IR light were significantly less likely to be normally distributed than sighted homing bouts under white light (*p* = 0.0063). See the supplement for observed and expected frequency counts, and all pairwise comparisons.

#### Lateral displacement

Mean lateral displacement (in cm) from the HV was greatest for sighted scorpions under red light (*M* = 12.79, SE = 1.58), and smallest for sighted scorpions under white light (*M* = 7.68, SE = 1.27). Blind scorpions had an intermediate lateral displacement (*M* = 8.70, SE = 3.04). An ANOVA (*n* = 34) was performed according to visual and light conditions, and approached significance, *F*(2, 31) = 2.68, *p* = 0.085 (Fig. 6d). A pairwise Tukey comparison found that the difference between lateral displacement in sighted scorpions under white light and under red light approached significance, *t* = − 2.18, *p* = 0.089. On average, scorpions strayed 10.41 cm (SE = 1.09) to either side of the HV.

#### Distance efficiency

Blind scorpions had the greatest SI (*M* = 0.90, SE = 0.11), while sighted scorpions under red light had the lowest (*M* = 0.62, SE = 0.08) (Fig. 7b). Sighted scorpions under white light produced an intermediate SI (*M* = 0.77, SE = 0.07). An ANOVA (*n* = 34) on the mean SIs according to visual and lighting conditions revealed no overall significance or pairwise comparisons at the 95% confidence interval, *F*(2, 31) = 2.30, *p* = 0.12. The overall mean SI was 0.72 (SE = 0.05).

## Discussion

In the present work, we provide a detailed analysis of homing movements in the scorpion *Mesobuthus eupeus*. Naïve scorpions are capable of returning to a shelter object in a manner that is directionally consistent with the direct path. The first-occurring homing bouts are characterized by paths consisting of turns about 10 cm to either side of the straightest home path and a distance efficiency of roughly three-quarters of the maximum efficiency. Altogether, *Mesobuthus eupeus* is capable of direct and seemingly deliberate homing behavior. In the following, we will evaluate the trial success as well as the applied methodology, and will hypothesize which sensory cues are involved in the homing behavior observed in *Mesobuthus eupeus*.

### Trial success and methodology

An important goal of our study was to develop a sensitive and convenient method for studying scorpion navigational behavior in the laboratory. It appears that our setup is successful at doing so. Beyond the convenience of data acquisition, this setup also reduces the confounding influence of human presence on scorpion behavior. Remote monitoring of trial progress through webcams removed the need to have an experimenter present in the room during trials, thereby preventing the disturbance of the scorpion. Since scorpions are especially sensitive to mechanical stimuli (Brownell 1977; Brownell and Farley 1979a, b, c), even small vibrations from human presence can disrupt the scorpions’ activity (from preliminary observations). The size of the arena used here provides the greatest navigational challenge in terms of distance (67.5 cm) out of all known laboratory studies of scorpion navigation. Compared with navigation over distances of roughly 4–6 (Bost and Gaffin 2004) or 5–7 (Vinnedge and Gaffin 2015) body lengths of the similarly sized *Paruroctonus utahensis*, our setup shows navigation over distances of 13–17 body lengths. The main advantage of the setup, however, is that direct handling of the scorpions before testing is not necessary. As discovered in preliminary observations, manually handling and moving scorpions stresses them and causes a defensive response or frantic movements. Other studies of scorpion navigation have involved manual displacement, and might therefore be affected by non-directed escape responses (e.g., Bost and Gaffin 2004). In contrast, scorpions here were allowed to exit the fauna box through the ramped door at will, and were already well-acclimated to the fauna box. As a result, the setup provides one of the least intrusive methods for studies of this type so far, and we can be sure that the subjects’ movements were not merely a panic response to handling.

A common obstacle in scorpion behavioral research is the apparent difficulty in motivating scorpions to participate. For example, Gaffin and Barker (2014) and Bost and Gaffin (2004) both reached a 45% overall success rate in their experiments involving scorpion locomotion and homing, respectively. The high failure rate of circadian control trials in the light stimulus experiment of this study is probably due to the time at which trials occurred. In the field, scorpion surface activity generally declines as dawn approaches (Fet 1980; Fleissner and Fleissner 2001a; Warburg 2013), so the scorpions were probably not motivated to begin exploring the arena only 3 h before imposed dawn. This trial condition was intended to compare the effect of a light stimulus at different time points, but unexpectedly confirmed the strong circadian activity rhythms already described in scorpion research (Fleissner and Fleissner 2001a). Consequently, future studies need to be aware of this circadian effect. Interestingly, the blind trials and infrared trials were much more likely to succeed than to fail with respect to trial validity, and were much more successful than all other trial conditions—and equally so. This could be explained by either the unavailability of visual scene information or reduced perceived light intensity upon departure from the box (see below, “Vision”). Our results show that the lighting configuration in terms of intensity and spectral composition plays an important role in the setup of behavioral assays and that infrared light is the best choice to perform meaningful experiments.

### Mechanism of navigation

#### Vision

Our results demonstrate that direct homing of *Mesobuthus eupeus* requires no visual information such as landmarks, panoramas, moonlight and starlight, the sky polarization compass, or optic flow. Similar to harvestmen (Silva et al. 2018), another arachnid, scorpions probably incorporate vision in homing when available, but in its absence rely heavily on path integration based on proprioception (see “Mechanosensation” section below).

On every measure of homing directness, visually impaired scorpions under white light performed most similarly to sighted scorpions under white light. Compared with sighted homing bouts under red light, sighted white light returns were significantly more likely to be directionally consistent (Fig. 5) and somewhat less displaced to either side of the HV (Fig. 6). Overall, it seems that there is a trend toward more direct homing bouts under brighter conditions, regardless of visual capacity. Perceived light intensity might, therefore, play a more important role in influencing path characteristics than visual scene information. Ecologically, the motivation to return to a known shelter more directly under bright light makes sense—scorpions might be more vulnerable to predation under brighter illumination. The implication that scorpions with painted eyes are still sensing ambient light is supported by the literature concerning extraocular photosensors (Zwicky 1968, 1970a, b; Rao and Rao 1973), and diminished light could also be reaching retinal photosensors through the paint. If ambient light influences the directness of homing as the data here suggest, eye paint apparently has little effect on perceived light intensity while under bright white illumination.

Perception of the red light intensity, on the other hand, may be diminished by eye paint as the high success rate of blind trials suggests. It has long been thought that scorpions are insensitive to red light (> 675 nm) (Machan 1968; Fleissner and Fleissner 2001b), although recent research disputes this claim (Roldan and Gaffin 2018). However, the light applied during departures from the box was not true red light and had peaks well within the range of green light (approximately 520–565 nm) and blue light (approximately 445–520 nm) (see “Apparatus” section). Since scorpions’ eyes are highly sensitive to green light (Machan 1968), the red light applied during departures was probably detectable. The eye paint would consequently have reduced the perceived brightness of the red ambient light stimulus, and thereby reduced a light avoidance response. This conclusion is supported by the similar results obtained from blinded animals and sighted animals under infrared light, which should not be detectable by the scorpions (Machan 1968; Fleissner and Fleissner 2001b). Regardless, the scorpions should have been less sensitive to the lower intensity red light than to the white light, which could explain the persistence of a behavioral response to white light despite the eyes being covered.

Compared to departure bouts of sighted animals under red light, homing bouts under white light were more likely to be directionally consistent regardless of vision. Departure bouts under IR were also less directionally consistent than sighted homing bouts under white light (see Fig. 5e). Interestingly, the effect disappears when scorpions depart from the box with their eyes covered, such that there is no difference in directional consistency between blind scorpions departing from the box and any homing scorpions. Therefore, the greater directional consistency of blind departures could be due to a loss of the ocular visual sense. Other arthropod navigators are known to gather visual scene information to compensate for potential errors or inconsistencies in path integration (e.g., Zeil et al. 1996; Nicholson et al. 1999; Wehner 2003; Nørgaard et al. 2012). The tortuous nature of sighted departures under red light could reflect scorpions’ attempts to familiarize themselves with the landmarks or panorama surrounding the box; in the absence of sight, scorpions may forgo this visual information gathering. Along these lines, sighted scorpions under red light—which is probably detectable by the scorpions—may also investigate visual objects on their way home, similar to how homing ants are distracted by novel visual information near the nest (Buehlmann et al. 2018), thereby explaining the indirectness of sighted red light returns compared with blind returns. If this is the case, exploratory behavior apparently may decrease under bright illumination. Additional trial conditions—such as sighted homing bouts under IR—could parse out the effects of light perception and vision loss on path characteristics, as well as inherent differences between outbound exploratory movements and directed homing.

#### Chemosensation

Scorpions possess an elaborate chemosensory system, with multitudinous chemosensory sensilla distributed over the entire body, concentrated on the tarsi, pedipalps, and pectines (see “Introduction and review” and Fig. 1). It has been proposed that scorpions can retrace their own paths using contact autochemosensation, or recognize chemical gradients in the area surrounding their burrows (Gaffin and Brayfield 2017). Our detailed analysis of departures and homing bouts shows no correlation between them in the sense of retracing a previous path (Fig. 4), and exclude autochemosensation as an important sense in homing over considerable distances, at least in the present species and experimental conditions. We found it unlikely that the scorpions followed substrate-borne chemical gradients back to the shelter; some individuals exhibited successful homing after an outbound journey which only crossed the arena once and in a rather straight path (see Supplementary Fig. 1), which probably does not allow sufficient familiarization with chemotextural gradients.

The role of airborne chemical stimuli in scorpion homing remains an open question. Homing by airborne olfactory cues has recently been demonstrated in a whip spider, another arachnid (Bingman et al. 2017; Wiegmann et al. 2019), which has modified antenniform first legs. Although we have no data that exclude olfaction as a factor in the homing abilities of scorpions, we classify this hypothesis as very unlikely for the following reasons: First, an elaborate sense of aerial olfaction in scorpions is still under debate and has only been suggested in predator avoidance (Nisani et al. 2018). Second, the habitat of desert scorpions, and thus the studied species here, is not in favor of olfactory cues (and chemosensory trails in general), as these would be quickly destroyed by the dry and hot conditions (Ruano et al. 2000).

Scorpions might also use their chemical sense to orient toward water, and could potentially locate areas of moist substrate near the burrow entrance (Abushama 1964; Gaffin et al. 1992). Even so, we have not applied such a gradient in our setup, which again leaves this potential mechanism unlikely. However, a chemosensory cue in the near field of the shelter might be possible. In this fashion, it is known that ants detect the exact location of their nest entrance by short-distance chemosensory cues (Steck et al. 2009).

#### Mechanosensation

The hypothesis of retracing textural gradients and one’s own footprints with mechanosensory sensilla (Gaffin and Brayfield 2017) can be refuted for the same reasons as autochemosensory trail retracing (see above). None of the scorpions followed their departure path back exactly in the homing bout (see Supplementary Figure S1).

Mechanosensory hairs called trichobothria on the pedipalps allow scorpions to use the horizontal wind direction to orient themselves in a process called anemotaxis (Hoffmann 1967; Linsenmair 1968, 1972; Fleissner 1977a, b; Krapf 1987; Meßlinger 1987; Fleissner and Fleissner 2001a). However, some scorpions live in environments that create swirling, unpredictable wind currents that probably make anemotaxis impossible, and they are nonetheless able to navigate (Polis et al. 1986). Additionally, no wind has been applied in our setup. Due to the heavy curtains surrounding the arena and its high walls, we exclude unexpected airstream influences.

Basitarsal compound slit sensilla and sensory hairs on the tarsal leg segments allow sand-dwelling scorpions to locate the source of vibrations from prey (Brownell 1977; Brownell and Farley 1979a, b, c). Whether the vibrational sense can be used to find objects such as shelters is unknown, and preliminary examinations of seismic echolocation have been inconclusive (Stephens 2000). Furthermore, due to the nature of vibrational signals in sandy areas, this sense would only be advantageous in the immediately adjacent environment of the animal, as vibrational waves can be detected only over a few decimeters (Brownell and van Hemmen 2001).

One mechanosensory aspect which is rather uninvestigated is proprioception. The leg segments of scorpions possess slit organs, which are known to function as proprioceptors in spiders (Seyfarth and Barth 1972; Seyfarth et al. 1982). Their function in scorpions has not been addressed with the exception of basitarsal slit organs (vibration, see above). Proprioception, which provides the animal with memorizable information about its own previous movements, is known to be used by several arthropods to calculate their position in a form of navigation called path integration (e.g., Görner 1958; Müller and Wehner 1988; Wittlinger et al. 2006; Kim and Dickinson 2017). Although no functional evidence for this idiothetic way of orientation is currently available for scorpions, it has been shown that the slit organs of the wandering spider *Cupiennius salei* play an important role in homing behavior: ablation of these organs led to drastic reduction in successful homing trials (Seyfarth and Barth 1972; Seyfarth et al. 1982). Recently, path integration has been proposed to play an important role in the homing of harvestmen (Silva et al. 2018).

## Conclusions and outlook

As in many systems, the homing ability of scorpions is probably based on a combination of several mechanisms, depending on the environmental conditions (Hoinville and Wehner 2018). As more information can be integrated, the more precise the behavioral response might be. Here, we added considerable support for directed linear movement towards the home shelter, and analyzed scorpion movement in detail. Our results suggest that path integration based on proprioception plays a crucial role for orientation and navigation. Furthermore, we present a methodological setup which allows the realization of diverse experiments dealing with behavioral questions, e.g., locomotion, activity patterns, shelter choice, or mating.

Although our results show that vision is not necessary for homing, the effects of lighting and visual conditions on the directness of movement are crucial for the performance of scorpions in behavioral assays, but not yet fully understood. Thus, it will be necessary in future studies to address the influence of different light intensities and spectral compositions. Interestingly, the sensory mechanisms involved in homing differ between chelicerate species. In whip spiders for example, olfaction and vision are crucial (Bingman et al. 2017), whereas proprioception and vision are involved in harvestmen (Silva et al. 2018). Homing trials of blinded scorpions under dim red light and of sighted scorpions under IR light may reveal whether illumination or vision is the more important factor. Clear paint applied to the eyes would probably obstruct retinal image formation and optic flow while still allowing high-intensity light to reach the eyes. By ablating the eyes, we could remove the ocular photoreception and visual effects completely to further examine the extraocular photosensory influence on homing.

Our pioneering study provides the first evidence that path integration is of pivotal importance for homing in *Mesobuthus eupeus*. As such, our work paves the way for the systematic study of other navigational senses. The next logical step is the detailed neuroanatomical description and ablation of slit organs to investigate proprioceptive cues and their potential role in path integration (Seyfarth and Barth 1972; Seyfarth et al. 1982). Displacement studies can give further information about scorpion HVs and search strategies related to path integration mechanisms.

## Electronic supplementary material

Below is the link to the electronic supplementary material.

Supplementary file1 (XLSX 64 kb)

Supplementary file2 (PDF 2602 kb)

## Data Availability

All relevant data generated and analyzed during this study are included in this published article and its supplementary information files. Video sequences and associated tracking coordinates generated with the software EthoVision XT 13.0 are available from the corresponding author upon reasonable request.
