# Supplementary material for: Non-visual homing and the current status of navigation in scorpions
Source: Anim Cogn. 2020 Apr 29;23(6):1215–34. doi: 10.1007/s10071-020-01386-z (PMC7700070; doi:10.1007/s10071-020-01386-z)
Supplement: Supplementary file 2 — Supplementary file2 (PDF 2602 kb) [file 10071_2020_1386_MOESM2_ESM.pdf]

Supplementary Figures associated with the following publication:

Title:

Non-visual homing and the current status of navigation in scorpions

Journal:

Animal Cognition

Authors:

Emily Danielle Prévost and Torben Stemme

Affiliations:

University of Ulm, Institute of Neurobiology, Albert-Einstein-Allee 11, 89081 Ulm, Germany

Corresponding author:

Torben Stemme

torben.stemme@uni-ulm.de

ORCID: 0000-0003-2751-2690

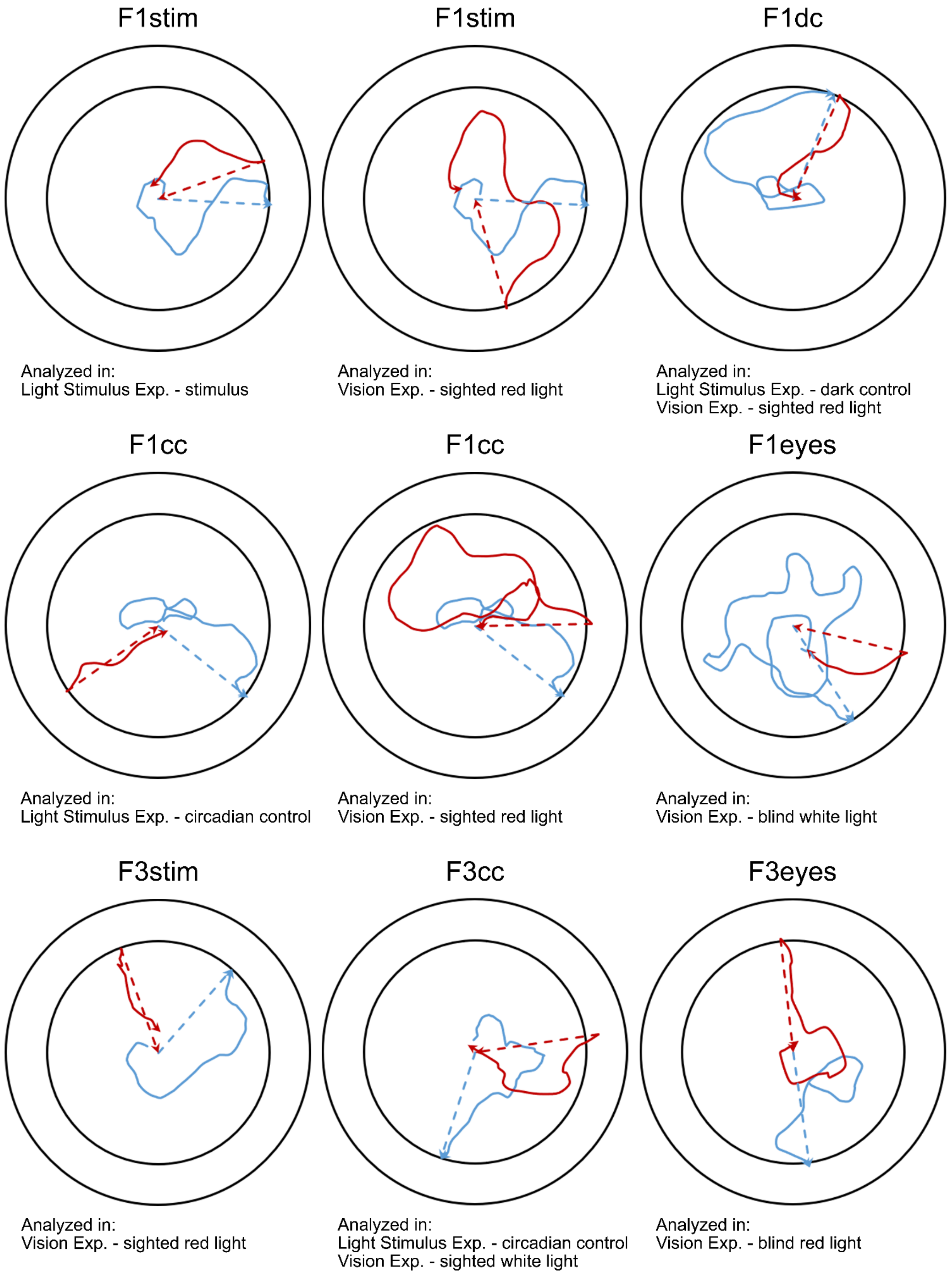

Fig S1I Depiction of all departures (solid blue lines) and homing bouts (solid red lines) and their respective vectors (dotted lines), which have been analyzed in the present study (continued in Figs S1II-VI, see below). Labels represent animal IDs and tested condition. Experiments in which trials have been analyzed are stated beneath each drawing. Abbreviations: cc, circadian control; dc, dark control; eyes, eyes covered by paint; F, female; IR: infrared; M, male; stim, stimulus

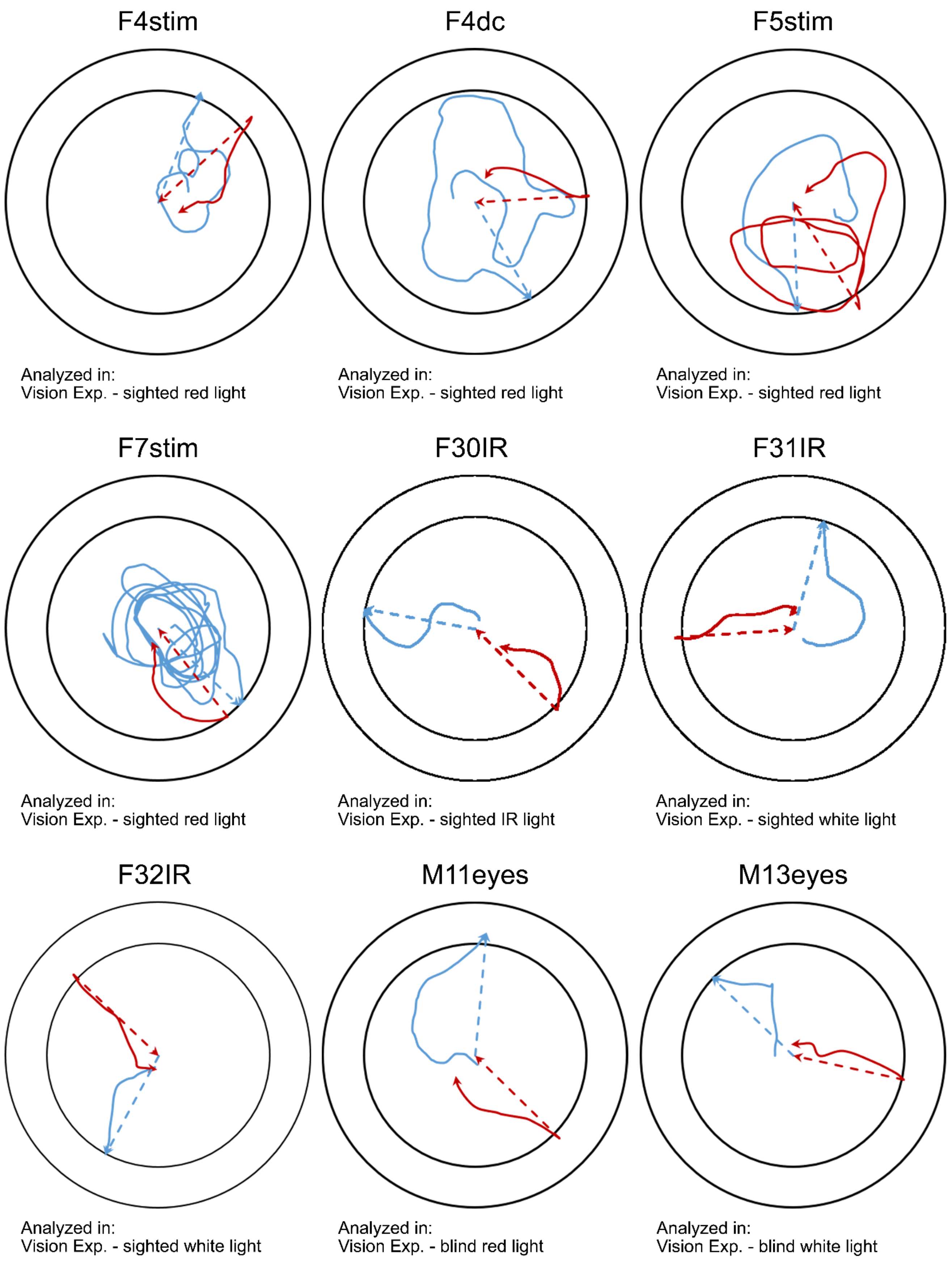

Fig S1II Continuation of Fig S1I

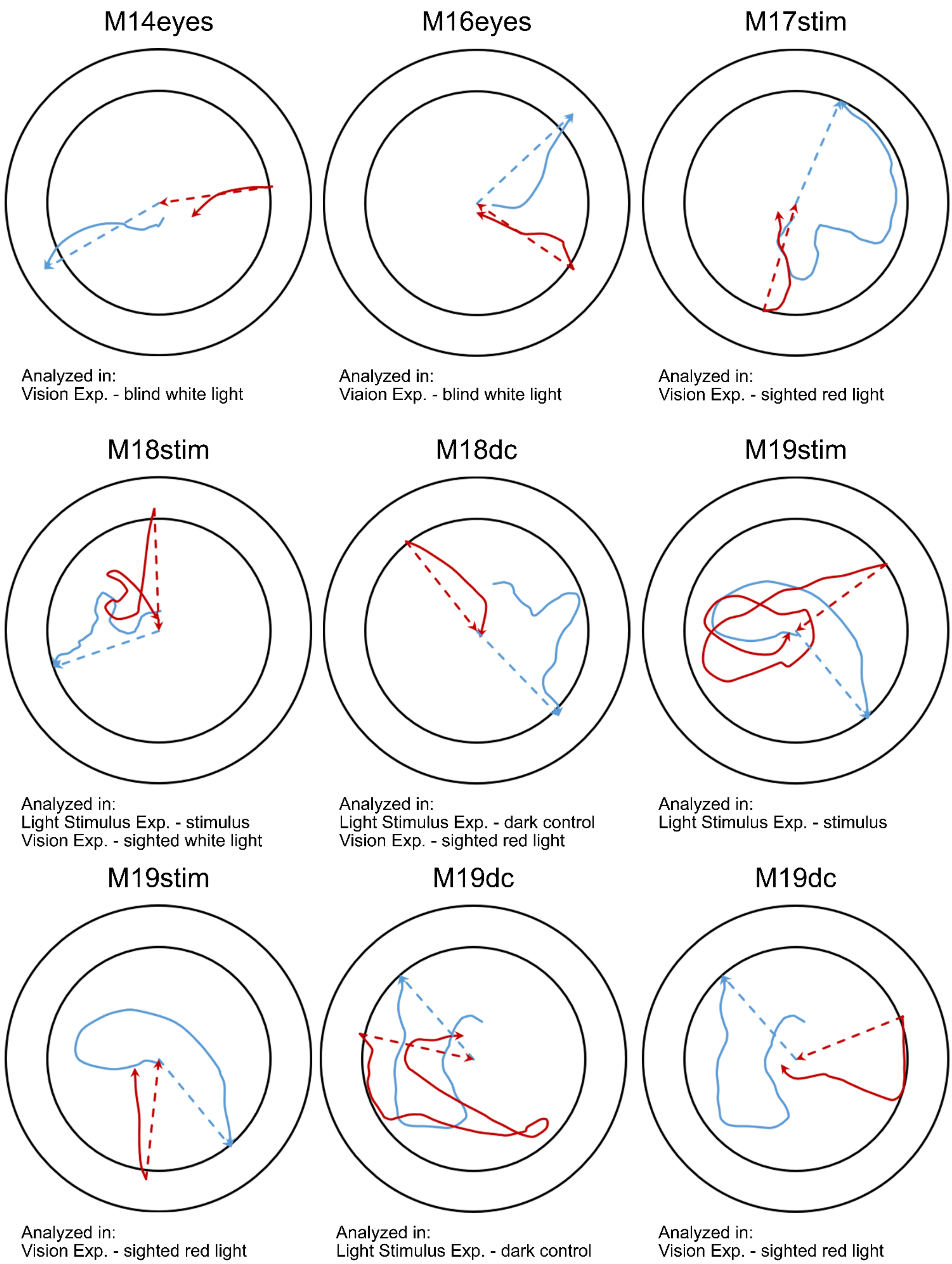

Fig S1III Continuation of Fig S1I

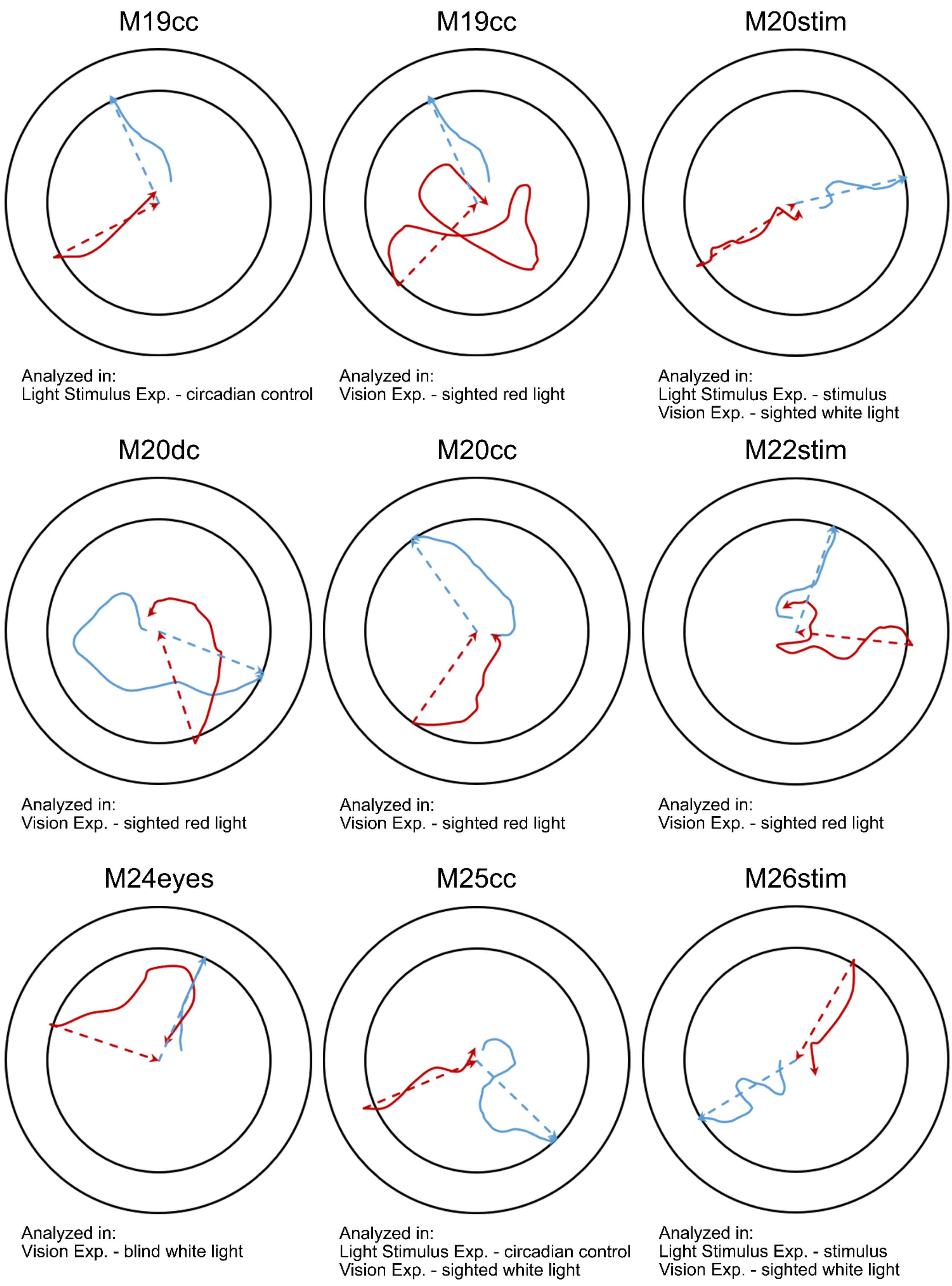

Fig S1IV Continuation of Fig S1I

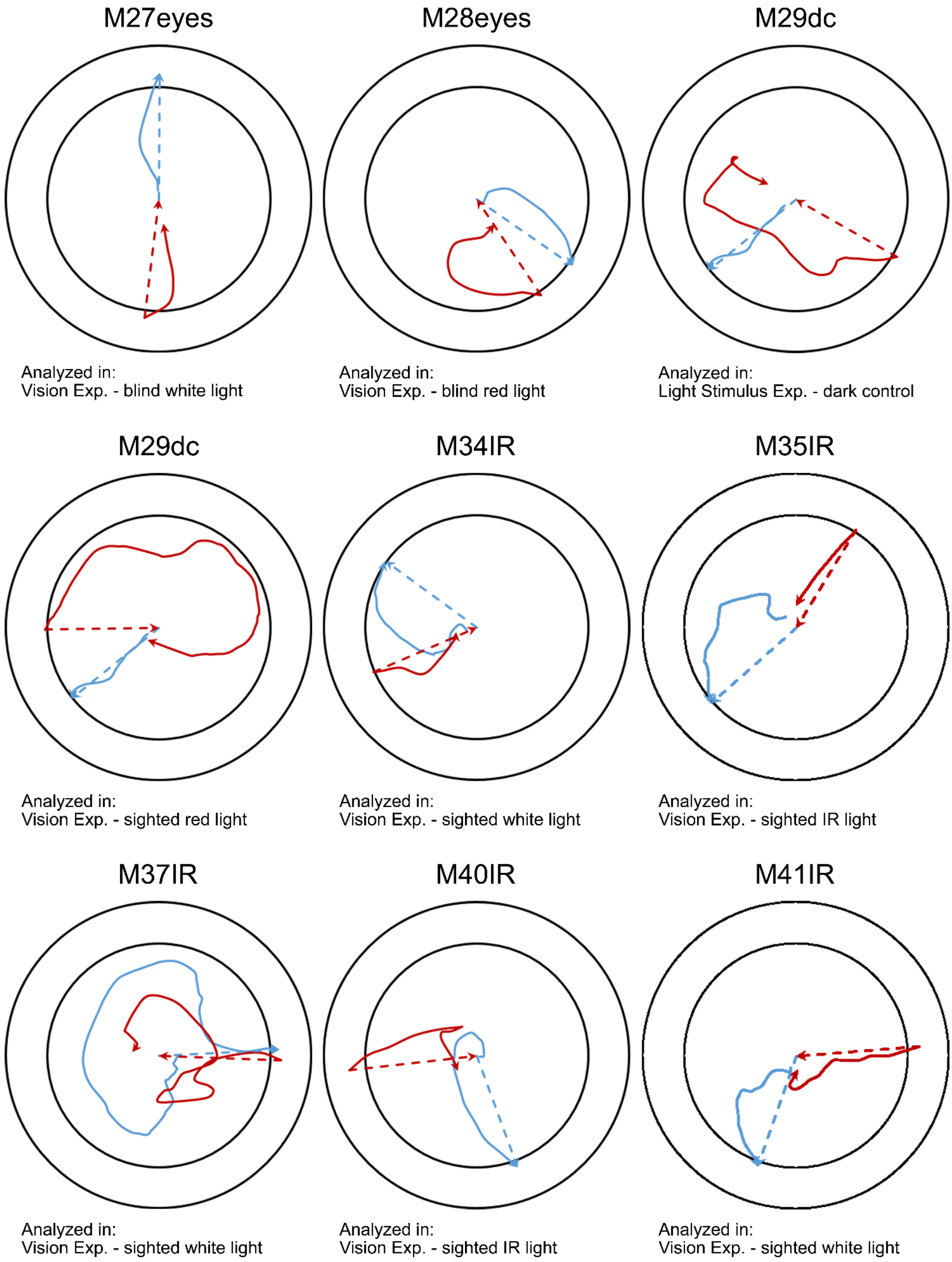

Fig S1V Continuation of Fig S1I

M48IR

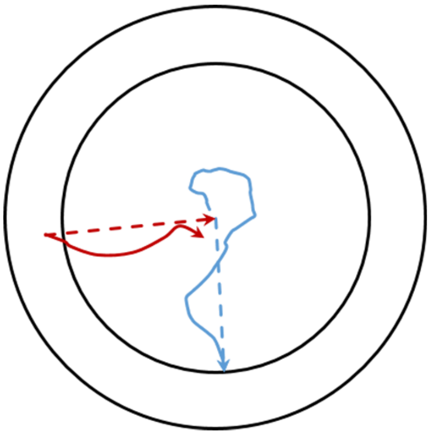

Analyzed in:
Vision Exp. - sighted white light

Fig S1VI Continuation of Fig S1I
